# Supplementary material for: Content-rich biological network constructed by mining PubMed abstracts
Source: BMC Bioinformatics. 2004 Oct 8;5:147. doi: 10.1186/1471-2105-5-147 (PMC528731; doi:10.1186/1471-2105-5-147)
Supplement: Additional File 2 — The original results of the above study (non-essential files are deleted to keep the file size under the limit set by BMC bioinformatics). [file 1471-2105-5-147-S2.bz2 › chilibotAdditionalFile2/dip05/515ID8799122E10818/html/ESR1_CREBBP.html]

 


 **ESR1** and **CREBBP** 
  
Found 57 abstracts in PubMed, retrieved 05.  
 

 What does Google say? 
 PDF only 
| .edu only 

---

**Interactive relationship** (e.g. stimulation, inhibition, etc)

**Stimulatory relationship**- Further, 4EM mediated transcription in ER alpha  [ **ESR1** ] , like estrogen, was enhanced in the presence of coactivators, steroid receptor coactivator 1 SRC 1, CREB binding  [ **CREBBP** ]  proteins CBP  [ **CREBBP** ] , and E6 associated protein E6 AP.  Ref: 12732288 J Steroid Biochem Mol Biol, 2003
**Neutral relationship**- Ligand Independent Interactions of p160 SRC Coactivators and CBP  [ **CREBBP** ]  with Estrogen Receptor alpha  [ **ESR1** ]  Regulation by Phosphorylation Sites in the A B Region Depends on Other Receptor Domains.  Ref: 12714702 Mol Endocrinol< MedlineTA>Mol Endocrinol, 2003
